# Histological Pattern of Endometrial Biopsies in Women with Abnormal Uterine Bleeding in a Hospital in North Central Nigeria

**DOI:** 10.1155/2018/2765927

**Published:** 2018-11-01

**Authors:** Ifeyinwa Mary Asuzu, Olaejirinde Olaniyi Olaofe

**Affiliations:** ^1^Department of Pathology and Forensic Medicine, University of Abuja, Abuja 902101, Nigeria; ^2^Department of Morbid Anatomy & Forensic Medicine, Obafemi Awolowo University, Ile-Ife 220282, Nigeria

## Abstract

The study is a retrospective cross-sectional study carried out in the Department of Pathology of Premier Hospital, Abuja, on specimens received over a one-year period. Four hundred and eighty-six samples of endometrial biopsies and curettings from women presenting with abnormal uterine bleeding sent to the histopathology laboratory were analyzed. The most common biopsies were those of product of conception which accounted for 304 cases (62.6%). Most of the cases of endometrial hyperplasia were typical. Endometritis and chorioamnionitis were the inflammatory conditions seen. Twenty-three women had molar pregnancies. The most common cause of abnormal uterine bleeding in this population is retained products of conception. There may be need to retrain some of the staff involved in the management of pregnancy related complications. There is need to further evaluate pregnancy related complications to ascertain the causes and circumstances responsible for them so as to appropriately direct interventional protocols.

## 1. Introduction

Endometrial biopsies are obtained for a number of reasons that include abnormal uterine bleeding in certain age groups, incomplete abortions, or suspected neoplasia and the endometrium may be sampled prior to certain procedures to treat infertility to determine the phase of the cycle to guide further tests or treatments [1].

The protocol to handle any endometrial sampling material is guided by the clinical indication for the specimen submission which may be for evaluation of infertility or preparation for In Vitro Fertilization (IVF), evaluation of abnormal uterine bleeding, and follow-up of a previous cytological or histological diagnosis [2].

The endometrium may be examined as part of a hysterectomy specimen and may be the site of a primary or secondary neoplastic process.

Cases of DUB may have structural or functional causes depending greatly on the age of the patient and clinical history [1].

Some studies showed infertility as the most common indication for endometrial biopsy and secretory phase endometrium as the commonest morphologic pattern encountered [3].

A low prevalence of tuberculous endometritis and endometrial carcinoma was also noted in similar studies [3].

Other studies reported endometrial hyperplasia as the most common morphological pattern encountered as well as a good number of endometritis cases though it was not said if endometrial culture was carried out in these cases [4]. Endometrial hyperplasia which is an intraepithelial nonneoplastic proliferative lesion was said to peak around the perimenopausal and menopausal period [5] with variable incidence in other studies [6, 7].

Lesions like endometrial polyps were found among other causes to be the most common structural cause of abnormal uterine bleeding and were of particular importance in patients being considered for IVF [8].

Varied morphological patterns have been reported in various parts of our country and the world usually from tertiary health care centres which may serve as regional reference centres and results from these studies may project abnormally high number of cases. We aim to document findings from a one-year review of cases seen in a community group private practice that caters to both primary health concerns and some degree of specialized care.

## 2. Materials and Methods

The study is a retrospective cross-sectional study carried out in the Department of Pathology of Premier Hospital, Abuja, on specimens received over a one-year period (June 2016- July 2017).

Four hundred and eighty-six samples of endometrial biopsies and curettings from women presenting with abnormal uterine bleeding sent to the histopathology laboratory were analyzed. Samples were fixed in 10% formal saline and routinely processed and stained with H&E. Histopathological evaluation was done under light microscope and consensus diagnosis was reached by two general pathologists.

Patients were categorized into reproductive age groups (18-40 yrs), perimenopausal (41-50), and postmenopausal (>50yrs).

## 3. Results and Discussion

**Figure 1:**
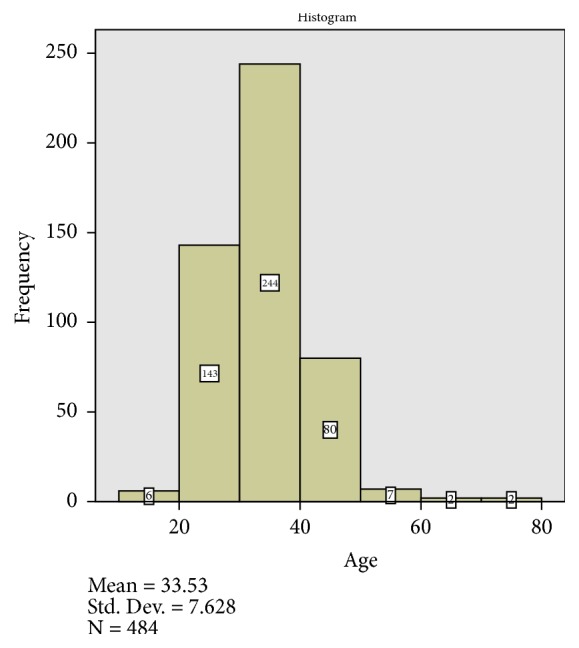
Age distribution of the study population. The age group of 30-40 years account for the highest number of cases.

A total of 486 endometrial biopsies were seen in Premier Hospital, Abuja, during the study period. The mean age of the patients was 33.53 yrs (SD=7.6). The age distribution of the study population is shown in Figure 1.

**Figure 2:**
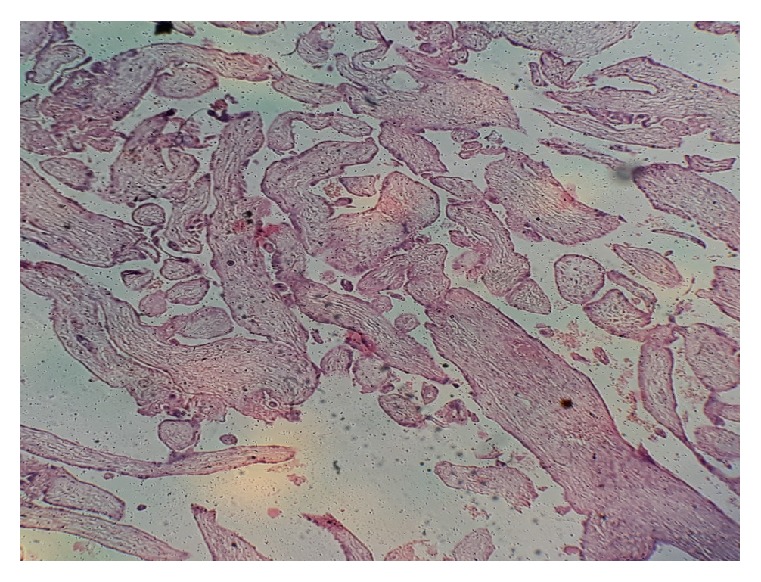
Products of conception (MVA). Chorionic villi in a 32-year-old woman who presented with bleeding per vaginam of 8 hours' duration.

The most common biopsies were those of product of conception which accounted for 304 cases (62.6%). A typical case with products of conception is shown in Figure 2. The next most common sample received was that of endometrial hyperplasia (49 cases, 10%). This was closely followed by endometrial polyp which accounted for 48 cases (9.9%). These are shown in Table 1.

**Figure 3:**
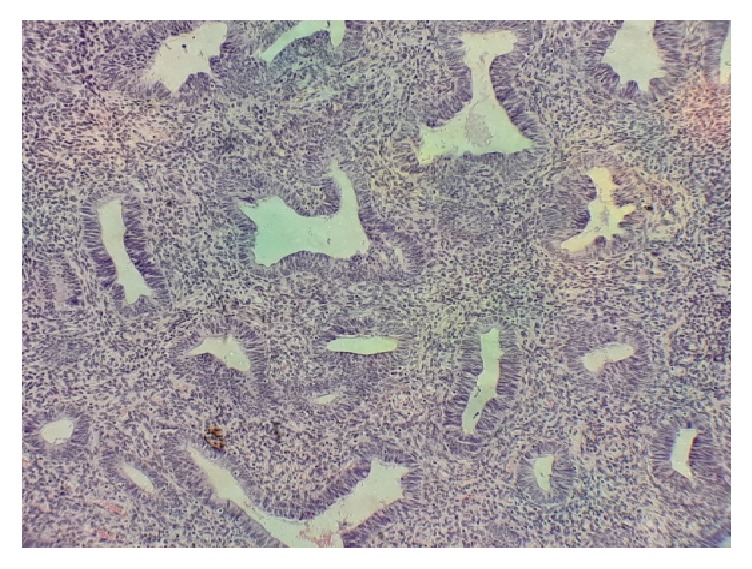
Simple typical hyperplasia. Typical hyperplastic glands in a 42-year-old woman who presented with bleeding per vaginam (intermenstrual).

The mean age of cases of endometrial hyperplasia was 38.3 years with a range of 22-62 years. Most of the cases of endometrial hyperplasia were typical (41cases). Majority (61%) of the typical endometrial hyperplasia fall in the age group of 18-40 years, while 13 cases (31.7%) were found in the range of 41-50 years. A case of simple typical hyperplasia is shown in Figure 3. Each of the age groups, 18-40 and 41-50 years, account for 4 cases of atypical endometrial hyperplasia.

**Figure 4:**
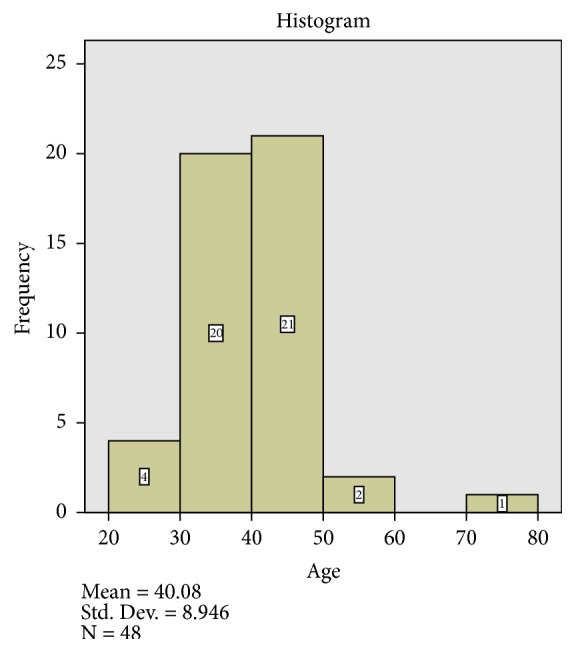
Histogram of cases of endometrial polyp. It shows the preponderance of cases of endometrial polyp in the age group of 30-50 years.

Most of the cases of endometrial polyp are found in the age group of 30-50 years which accounted for 41 cases (85.4%). Only 3 cases of endometrial polyp were found in women above 50 years of age. These are presented as a chart in Figure 4.

Endometritis and chorioamnionitis were the inflammatory conditions seen. Only four cases of chorioamnionitis were seen. Three of these were in the age group of 18-40 years. Of the seven cases of endometritis seen in this study, one was a case of tuberculosis seen in a 31-year-old woman with a clinical diagnosis of molar pregnancy.

Two cases of endometrial carcinoma were seen during the study period. The oldest was a 70-year-old woman while the youngest was 60 years old. They both presented with postmenopausal bleeding.

Calcified endometrial tissue was seen in two women aged 38 and 32 years. The two cases were associated with the presence of foreign body in the uterus.

**Figure 5:**
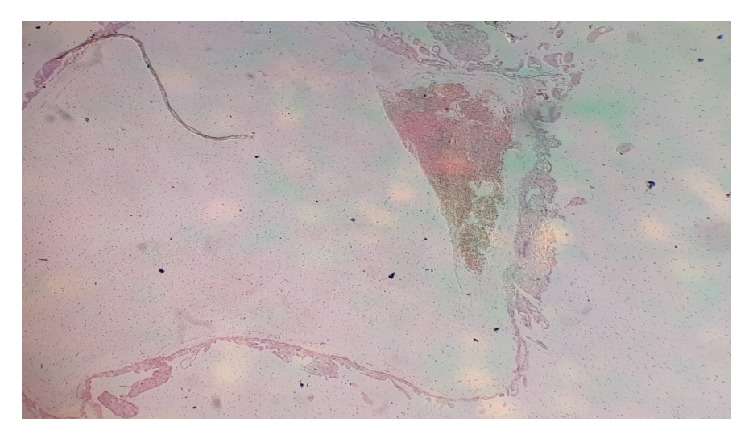
Hydropic villus in complete hydatidiform mole. A histologic section of hydropic villus in a 36-year-old woman presenting with vaginal bleeding and passage of grape-like vesicles per vaginam.

Twenty-three women had molar pregnancies. Ten of these were partial moles which were all found in women in the age group of 18-40 years. This age group also accounted for 12 cases of complete mole. Only one complete mole was seen in the age range of 41-50 years. The mean age of patients with partial mole was 30.3 years, while that of complete mole was 29.8 years. There was no significant association between the types of mole and the age groups (p=0.37). A case of complete hydatidiform mole is shown in Figure 5.

**Figure 6:**
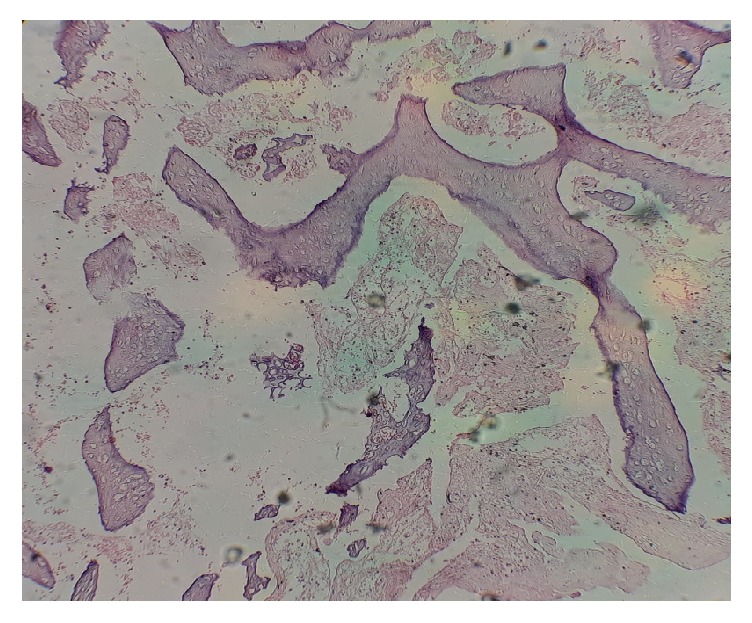
Osseous metaplasia. A histologic section of osseous metaplasia in a 29-year-old woman with primary infertility and history of missed abortion.

There were two cases of osseous metaplasia, all in the age group of 18-40 years. They were associated with the presence of foreign body within the uterus. A case of osseous metaplasia is seen in Figure 6.

Submucous leiomyoma was seen in two cases. The older and the younger patients were 44 and 41 years, respectively. They presented with abnormal vaginal bleeding.

Normal endometrium was found in 39 cases. Twenty-three was in proliferative phase and 16 in secretory phase. Most of these cases with normal biopsies were being managed for infertility.

## 4. Discussion

The study population mainly comprises of women with reproductive health conditions as the hospital is partly specialized in treatment of infertility. Expectedly, the vast majority of the patients fall within the main reproductive years 20-50 age group (467 cases). This is similar to report from a study by Ojo* et al.* from a tertiary center in the same region and studies in a tertiary center in India [1, 3].

The most common cause of abnormal uterine bleeding in this population is retained products of conception similar to reports from India [1] and Ilorin, Nigeria [3], and at variance with studies by Khan* et al*. (5.8%) [4]. This may be attributable to the relatively poor maternal health care in Nigeria, with dwindling resource allocation to health care. Many publications have identified the relatively high incidence of retained products of conception in Nigeria [3]. This can contribute to anaemia and morbidity in women [9]. It may be noteworthy to mention that many minor obstetric procedures are carried out by poorly trained paramedical staff and traditional birth attendants. There may be need to retrain some of these staff in management of such pregnancy related complications. There is also need to further evaluate these cases to ascertain the causes and circumstances responsible for them so as to appropriately direct interventional protocols. S vaidya* et al*. found normal endometrium to be the most common morphological correlate [10].

Endometrial polyp was found in the same frequency in patients above and below 40 years of age. Clinicians should be aware of this fact and should have a high index of suspicion of endometrial polyp in women older and younger than 40 years of age. The high prevalence of endometrial polyps in the patients in this study may not be unrelated to the fact that a number of them may have been on hormonals used for management of infertility. It is important to note that the relatively skewed age distribution may contribute to this finding.

About 39 cases had normal endometrium at either the proliferative or the secretory phases. This accounts for 8% of the cases and were cases of dysfunctional uterine bleeding (DUB). This is at variance with reports from Ilorin which revealed a high frequency of secretory phase endometrium (20.3%) [3] and studies from elsewhere in the world [11, 12]. Gynaecologists should endeavour to thoroughly investigate all cases and not be in a hurry to make diagnosis of DUB. Our study has shown presence of various pathologies in over 90% of cases.

Molar pregnancy was found in 23 cases (4.7%). This is similar to findings in Ilorin (1.5%) [3], due to the relative rarity of the lesion. Most of the patients were below 40 years of age. This is not surprising as the entity is associated with gestation in most cases. Although relatively uncommon, molar gestation should be suspected in all cases of abnormal uterine bleeding especially if there are markedly elevated serum human chorionic gonadotrophins.

Inflammatory conditions were found in 11 cases. Similar frequencies have been reported in Ilorin and some studies from Asia [3, 4, 10, 12], areas with similar socioeconomic conditions. Interestingly, one was a case of tuberculous endometritis. Tuberculosis though said to be endemic in Nigeria is relatively under control especially with the very successful directly observed therapy. It is also important to highlight the fact that the rarity of tuberculous endometritis could be explained by the rapid decline in incidence of disseminated tuberculosis in the country. The study centre is an urban area and may not explain findings in relatively rural settings. However, gynaecologists should be aware that disseminated tuberculosis can result in chronic endometritis with resultant abnormal uterine bleeding. This further reinforces the fact that exhaustive investigations should be carried out on every case of unusual uterine bleeding.

As has been shown in various reports, endometrial carcinoma was found in postmenopausal women [1, 3, 10]. Only two cases were seen in this study similar to reports from comparative studies [1, 3, 10, 11]. This low frequency can be attributed to the fact that the study population mainly consists of women of reproductive age.

## 5. Conclusions

The most common cause of abnormal uterine bleeding in this population is retained products of conception. There may be need to retrain some of the staff involved in the management of pregnancy related complications. There is need to further evaluate pregnancy related complications to ascertain the causes and circumstances responsible for them so as to appropriately direct interventional protocols. Although relatively uncommon, molar gestation should be suspected in all cases of abnormal uterine bleeding especially if there are markedly elevated serum human chorionic gonadotrophins.

## Figures and Tables

**Table 1:** List of various diagnosis and the corresponding age groups.

	18-40 (years)	41-50 (years)	>50 (years)
**INFLAMMATORY LESIONS**			

Acute endometritis	3 (75%)	1 (25%)	0 (0%)

Chronic endometritis	3 (100%)	0 (0%)	0 (0%)

Chorioamnionitis	3 (75%)	1 (25%)	0 (0%)

**PROLIFERATIVE NON-NEOPLASTIC LESIONS**			

Atypical Hyperplasia-	4 (50%)	4 (50%)	0 (0%)

Typical Hyperplasia	26 (63.4%)	13 (31.7%)	2 (4.9%)

Endometrial polyp	24 (50.0%)	21 (43.8%)	3 (6.3%)

**NEOPLASTIC LESIONS**			

Leiomyoma	0 (0%)	2 (100%)	0 (0%)

Partial mole	10 (100%)	0 (0%)	0 (0%)

Complete mole	12 (92.3%)	1 (7.7%)	0 (0%)

Endometroid carcinoma	0 (0%)	0 (0%)	2 (100%)

**NORMAL ENDOMETRIUM**			

Proliferative phase	17 (73.9%)	6 (26.1%)	0 (0%)

Secretory phase endometrium	13 (75%)	2 (25.0%)	1 (0%)

**OTHERS**			

Products of conception	286 (94.1%)	18 (5.9%)	0 (0%)

Calcified endometrial tissue	2 (100%)	0 (0%)	0 (0%)

Osseous metaplasia	2 (100%)	0 (0%)	0 (0%)

Stromo-glandular dissociation	1 (33.3%)	2 (66.7%)	0 (0%)

Inconclusive	0 (0%)	1 (100.0%)	0 (0%)

TOTAL	406 (83.5%)	72 (14.8%)	8 (1.6%)

## Data Availability

The data used to support the findings of this study are available from the corresponding author upon request.
